# EdgeOpt-Sched-CS: Cold-Start-Aware Dynamic Scheduling for Efficient DNN Inference at the Edge

**DOI:** 10.3390/s26103130

**Published:** 2026-05-15

**Authors:** Yuchang Gu, Diming Zhang, Taiyu Lu

**Affiliations:** 1 Ocean College, Jiangsu University of Science and Technology, Zhenjiang 212000, China; 242212288918@stu.just.edu.cn (Y.G.); 252212288222@stu.just.edu.cn (T.L.); 2 School of Computer, Jiangsu University of Science and Technology, Zhenjiang 212000, China

**Keywords:** edge inference, operator scheduling, cold-start optimization, dynamic scheduling, graph neural networks, warm-start scheduling, transfer learning

## Abstract

Dynamic scheduling can improve the latency and memory efficiency of deep neural network inference on edge devices, but it often introduces cold-start overhead when a newly deployed model requires online profiling and policy adaptation before reaching stable performance. This paper proposes EdgeOpt-Sched-CS, a cold-start-aware extension of dynamic graph scheduling for edge inference. The key idea is to initialize the scheduler of a target computation graph using scheduling knowledge transferred from structurally similar source graphs, instead of starting from a generic policy. EdgeOpt-Sched-CS constructs compact graph signatures, retrieves relevant source schedulers, and performs lightweight cold-start-aware online adaptation during early deployment. We evaluate the framework across representative device–model scenarios involving lightweight convolutional neural networks, transformer models, and quantized language-model workloads. The results show that EdgeOpt-Sched-CS reduces cumulative cold-start latency by 10.6–20.4% and shortens time-to-stability by 5.2–21.7%, while preserving the steady-state latency–memory behavior of the original dynamic scheduler with only small additional scheduling overhead. These findings indicate that scheduler initialization is an important optimization dimension for adaptive edge inference and that prior scheduling knowledge can be effectively reused across related computation graphs.

## 1. Introduction

Edge artificial intelligence (Edge AI) is increasingly reshaping latency-sensitive applications such as autonomous perception, intelligent surveillance, industrial control, and real-time human–machine interaction. In these scenarios, inference must be executed close to the data source under strict constraints on computation, memory bandwidth, energy consumption, and thermal budget. As deep neural networks (DNNs) continue to grow in scale and structural complexity, efficient deployment on resource-constrained edge devices has become a central systems challenge. Recent surveys have shown that edge inference performance is jointly determined by model architecture, compiler support, runtime scheduling, and hardware-aware adaptation, rather than by model compression alone [1,2,3,4].

Intermediate representations (IRs) and deep learning compilers have become a standard foundation for portable deployment. Open Neural Network Exchange (ONNX) provides a common IR for model interoperability across frameworks and runtimes [5], while compiler systems such as Tensor Virtual Machine (TVM), Relay, Glow, Multi-Level Intermediate Representation (MLIR), nGraph, and Tensor Comprehensions expose graph-level and operator-level optimization opportunities for heterogeneous hardware back-ends [6,7,8,9,10,11]. This line of work has significantly improved the deployability and performance portability of DNN inference. In parallel, compiler-oriented research has demonstrated that automatic scheduling and graph-level optimization can produce substantial performance gains through learned search, substitution rules, and tensor-program generation [12,13,14,15,16]. However, most of these approaches are primarily designed for offline or compile-time optimization, and therefore assume that execution conditions remain sufficiently stable after deployment.

Such an assumption is often violated on edge devices. In real deployment, runtime conditions fluctuate because of background contention, variable memory pressure, thermal throttling, and changing request patterns. Static execution plans, even when well optimized offline, may therefore become suboptimal once the actual deployment environment diverges from the assumptions used during compilation or tuning [1,16]. This problem becomes more pronounced for graph-structured workloads with heterogeneous operators and irregular memory behavior, such as transformer inference and compact large language models (LLMs) deployed under constrained edge resources [2,17].

To address this limitation, adaptive scheduling has emerged as a promising direction. In our setting, the baseline system, EdgeOpt-Sched, constructs an operator dependency graph (ODG), collects lightweight runtime telemetry, encodes graph structure with a graph neural network (GNN), and applies reinforcement learning (RL), specifically Proximal Policy Optimization (PPO), to select execution orders while coordinating memory reuse and graph-level execution decisions. This design is motivated by two well-established ideas: first, graph encoders, including graph convolutional networks (GCNs), GraphSAGE, graph attention networks, and graph isomorphism networks, are effective at capturing structural information in graph-structured decision problems [18,19,20,21]; second, policy-gradient and trust-region style RL methods such as PPO offer a practical balance between training stability and implementation simplicity for sequential decision-making under uncertainty [22,23,24,25].

Although dynamic scheduling improves steady-state execution quality, it introduces a different systems bottleneck: cold-start overhead. When a new model is deployed, the scheduler must still undergo a warm-up and online adaptation phase before reaching an effective execution policy. During this phase, telemetry is gathered, graph embeddings are formed, and the policy is refined based on early execution feedback. As a result, a scheduler that learns from scratch for every newly deployed graph pays a non-negligible deployment-time penalty before it can fully exploit its adaptive capability.

Cold-start overhead is particularly important in edge settings for three reasons. First, many edge workloads are not dominated by very long steady-state runs; instead, models may be loaded on demand, updated frequently, or invoked intermittently. In such cases, the latency observed during the first several inference requests directly affects user-perceived responsiveness. Second, larger transformer-style models and compact edge LLMs exhibit richer operator heterogeneity, deeper dependency chains, and stronger memory sensitivity, all of which amplify the cost of poor early scheduling decisions [3,17]. Third, from a systems perspective, evaluating only post-convergence latency can obscure an important part of the deployment lifecycle: the interval between model loading and the point at which the scheduler becomes reliably effective.

Motivated by transfer learning and fast-adaptation principles, a practical strategy for reducing cold-start cost is to reuse scheduling knowledge accumulated from previously optimized source models, rather than initializing the scheduler from a generic state for every newly deployed graph [26,27,28,29].

Hence, we propose EdgeOpt-Sched-CS, a cold-start-aware extension of EdgeOpt-Sched [30] for edge DNN inference. The framework augments the original dynamic scheduling pipeline with three mechanisms: model-signature construction, graph-signature-based retrieval from a source-model bank, and cold-start-aware online adaptation. Specifically, each computation graph is represented by a compact signature that summarizes its topology, operator composition, and coarse cost characteristics. This signature is used to retrieve structurally relevant source schedulers, whose parameters are then used for warm-start initialization on the target graph. During the early deployment window, the scheduler is further optimized with a cold-start-aware objective that explicitly prioritizes early-stage efficiency while preserving the long-term latency–memory optimization target of the base framework.

Compared with existing work, EdgeOpt-Sched-CS contributes a deployment-oriented perspective in two respects. First, it reframes scheduler initialization as a transfer problem over graph-structured workloads instead of treating every new deployment as an independent online-learning task. Second, it expands the evaluation target from steady-state efficiency alone to the full deployment lifecycle, including the early adaptation window. This distinction matters because a scheduler that is optimal only after many warm-up steps may still be undesirable in practice, particularly on edge devices serving bursty or intermittent workloads.

Although EdgeOpt-Sched-CS is related to transfer learning, meta-learning, and warm-start reinforcement learning, it differs from applying a generic fast-adaptation algorithm to scheduling. First, the transfer unit in our setting is a computation graph with explicit operator dependencies, memory behavior, and edge-device telemetry, rather than an abstract task sampled from a generic task distribution. Second, the initialization is target-dependent: the scheduler is initialized from a source graph retrieved by normalized and weighted graph-signature similarity, instead of relying only on a single global meta-initialization. Third, the optimization target explicitly includes deployment-onset metrics, including cumulative cold-start latency, time-to-stability, and cold-start regret, rather than only long-term return after adaptation. Therefore, the novelty of EdgeOpt-Sched-CS lies in integrating graph-structured retrieval, encoder–policy warm-start transfer, and cold-start-aware online scheduling into a deployment-oriented edge inference framework.

The main contributions of this work are summarized as follows:

- We identify cold-start overhead as a practical bottleneck in dynamic graph scheduling for edge DNN inference and formalize deployment-onset efficiency using cumulative latency, time-to-stability, and cold-start regret.
- We propose EdgeOpt-Sched-CS, a cold-start-aware scheduling framework that performs target-dependent warm-start initialization through graph-signature-based source retrieval.
- We design a compact graph signature that combines topology, operator composition, and coarse runtime-cost descriptors to support scheduling-relevant source selection across heterogeneous DNN graphs.
- We integrate warm-start encoder–policy transfer with cold-start-aware online adaptation, allowing the scheduler to reduce early deployment overhead while preserving the steady-state behavior of the original dynamic scheduler.
- We establish a cold-start-oriented evaluation protocol and show that EdgeOpt-Sched-CS reduces early cumulative latency and time-to-stability across representative edge deployment scenarios and unseen target graphs.

The remainder of this paper is organized as follows. Section 2 reviews the most relevant literature on IR-based optimization, graph-level DNN optimization, learning-based scheduling, and edge inference scheduling. Section 3 presents the proposed EdgeOpt-Sched-CS framework. Section 4 describes the experimental design and evaluation protocol. Section 5 reports the main results. Section 6 discusses implications, limitations, and future directions.

## 2. Related Work

Research most closely related to our work can be grouped into four directions: IR-based compiler optimization, graph-level DNN optimization, learning-based scheduling and fast adaptation, and edge inference scheduling.

### 2.1. IR-Based Compilation and Operator Scheduling

IR-based deployment frameworks have become a standard foundation for portable DNN inference. ONNX provides a common intermediate representation that enables interoperability across frameworks and runtimes [5]. On top of such IRs, compiler stacks such as TVM and Relay support graph lowering, schedule search, and backend-specific code generation for heterogeneous devices [6,7]. Other compiler infrastructures, including Glow, MLIR, nGraph, and Tensor Comprehensions, further show that high-level graph abstractions and low-level tensor optimization can be integrated into a unified deployment pipeline [8,9,10,11]. Comprehensive surveys confirm that compiler design has become one of the dominant factors in practical deep learning system performance [16].

Within this line of work, automatic schedule optimization has attracted significant attention. AutoTVM and Ansor demonstrate that tensor-program search can be guided using learned cost models and large search spaces to produce high-performance schedules automatically [12,13]. Similarly, the Halide autoscheduler shows that learned search strategies can generate efficient schedules for structured program optimization [15]. These methods have substantially advanced compile-time optimization, but they remain primarily offline methods. They do not directly address deployment-time cold-start behavior on edge devices, where the runtime environment may differ from the compile-time assumption.

### 2.2. Graph-Level Optimization for Deep Learning Computation

Another important line of work focuses on graph-level optimization beyond operator-local tuning. Tensor Algebra SuperOptimizer (TASO) automatically searches for graph substitutions and improved graph forms under a cost model, showing that graph rewrites can produce substantial gains beyond manually designed graph optimizations [14]. Related graph-level transformations also appear in compiler infrastructures such as Relay and Glow, where fusion, layout rewriting, and operator reorganization are essential to runtime performance [7,8]. These studies demonstrate that graph structure is an important optimization target in its own right.

Our work shares the view that graph structure matters, but the optimization problem is different. Instead of rewriting the graph offline, we focus on operator-level execution scheduling over an already deployed graph. More importantly, our emphasis is not on finding only a good steady-state schedule, but on reducing the latency penalty incurred before the scheduler becomes effective.

### 2.3. Learning-Based Scheduling and Fast Adaptation

Our framework is also related to learning-based optimization and adaptive scheduling. GNNs provide an effective way to encode graph-structured workloads and have become a standard representation model for dependency-aware decision problems [18,19,20,21,31]. Reinforcement learning has likewise been used to optimize sequential decisions in systems and combinatorial settings [22,23,24,25,32,33]. These two ideas together motivate the use of graph encoders plus RL for operator scheduling under dynamic runtime conditions.

A second relevant thread is meta-learning and fast adaptation. Model-Agnostic Meta-Learning (MAML), Reptile, and RL^2^ all seek parameterizations that support rapid adaptation to unseen tasks from limited experience [26,27,28]. Meta-RL surveys further show that fast adaptation is particularly valuable when online interaction is expensive or unstable [29]. Our method is conceptually related to this principle but differs in two ways. First, we address graph-structured operator scheduling rather than generic task adaptation. Second, instead of using only global meta-initialization, we introduce graph-signature-based retrieval from a source-model bank, which allows the initialization to depend explicitly on structural similarity between source and target graphs.

Compared with these general fast-adaptation methods, our setting introduces additional structure and constraints. The target task is not only to adapt a policy quickly, but to schedule operators in a directed computation graph under dependency, memory, and edge-device runtime constraints. Moreover, EdgeOpt-Sched-CS does not rely solely on a task-agnostic meta-initialization. Instead, it retrieves a source scheduler according to graph-signature similarity, which makes the initialization explicitly dependent on the topology, operator composition, and cost profile of the target graph. This graph-conditioned retrieval mechanism is the key distinction between the proposed method and generic meta-learning or warm-start reinforcement learning approaches.

### 2.4. Edge Inference Systems and Scheduling

A broad body of work studies edge inference from the perspective of model partitioning, compression, approximation, and runtime scheduling. Collaborative execution systems such as Neurosurgeon, Edgent, and JointDNN show that model partitioning between device and edge/cloud can significantly improve latency under resource constraints [34,35,36]. Other systems, including MCDNN, DeepThings, and NestDNN, focus on approximate execution, distributed edge inference, or multi-tenant on-device execution [37,38,39]. More recent surveys confirm that efficient edge inference depends on the joint design of hardware-aware optimization, compression, scheduling, and deployment strategy [1,2,3].

Model-level adaptation techniques are also highly relevant. BranchyNet, SkipNet, and BlockDrop reduce computation by dynamic early exit or conditional execution [40,41,42]. Compression and deployment-oriented adaptation methods such as Deep Compression, NetAdapt, AMC, HAQ, Once-for-All, MCUNet, and TinyTL show how pruning, quantization, architecture specialization, and lightweight fine-tuning can make inference practical on constrained devices [43,44,45,46,47,48,49]. These techniques improve model efficiency, but they do not directly solve the cold-start problem of graph execution scheduling after model deployment.

At the scheduling level, recent studies have begun to investigate inference workload management in edge and edge–cloud environments. For example, Castellano et al. consider RL-based inference workload scheduling in the computing continuum [50], while Sun et al. study adaptive scheduling of online inference pipelines at the edge [51]. Such works are highly relevant from a systems viewpoint, but they primarily operate at the level of request dispatching, pipeline organization, or node-level workload allocation. By contrast, our work addresses a finer-grained problem: operator-level scheduling within a deployed computation graph, with explicit emphasis on cold-start overhead.

### 2.5. Position of This Work

In summary, prior research has established strong foundations for IR portability, graph optimization, learned scheduling, model adaptation, and edge inference deployment. However, the specific problem of reducing *cold-start overhead in dynamic graph scheduling for edge DNN inference* remains insufficiently explored. EdgeOpt-Sched-CS addresses this gap by combining graph-signature-based retrieval, warm-start initialization, and cold-start-aware online adaptation in a single deployment-oriented framework. In this sense, our work complements prior compiler and edge systems research by focusing not only on how to make execution efficient eventually, but also on how to make it efficient quickly after deployment.

Beyond performance optimization, this problem is also relevant to industrial Internet of Things (IIoT) settings, where delayed model stabilization may postpone anomaly detection, fault diagnosis, or security monitoring decisions under strict timing requirements.

## 3. Method

### 3.1. Overview

EdgeOpt-Sched-CS is designed as a cold-start-aware extension of the original EdgeOpt-Sched framework. The original system already provides four essential ingredients for adaptive scheduling on edge devices: (i) operator dependency graph construction, (ii) lightweight online profiling, (iii) GNN-based graph encoding, and (iv) PPO-based scheduling with memory-aware execution planning. These components allow the scheduler to adapt to runtime conditions and outperform static execution strategies, but they also imply an unavoidable early-stage adaptation cost because the scheduler must refine itself after deployment on a new model.

Our goal is to reduce this early-stage cost without sacrificing steady-state quality. To this end, EdgeOpt-Sched-CS introduces three additional mechanisms on top of the original pipeline:

1. **Model Signature Construction**, which summarizes a computation graph using topology, operator composition, and coarse cost statistics;
2. **Graph-Signature-Based Retrieval and Warm-Start Initialization**, which identifies relevant source schedulers from a source-model bank and transfers their parameters to the target graph;
3. **Cold-Start-Aware Online Adaptation**, which emphasizes early-step latency reduction during the initial deployment window and then gradually returns to the original long-term optimization objective.

The resulting system keeps the original closed-loop scheduling design but replaces “generic initialization + full warm-up” with “graph-aware initialization + lightweight adaptation.” Conceptually, the framework no longer treats each newly deployed model as a cold start from scratch; instead, it views target-graph scheduling as an adaptation problem over a structured family of previously observed computation graphs.

**Figure 1:**
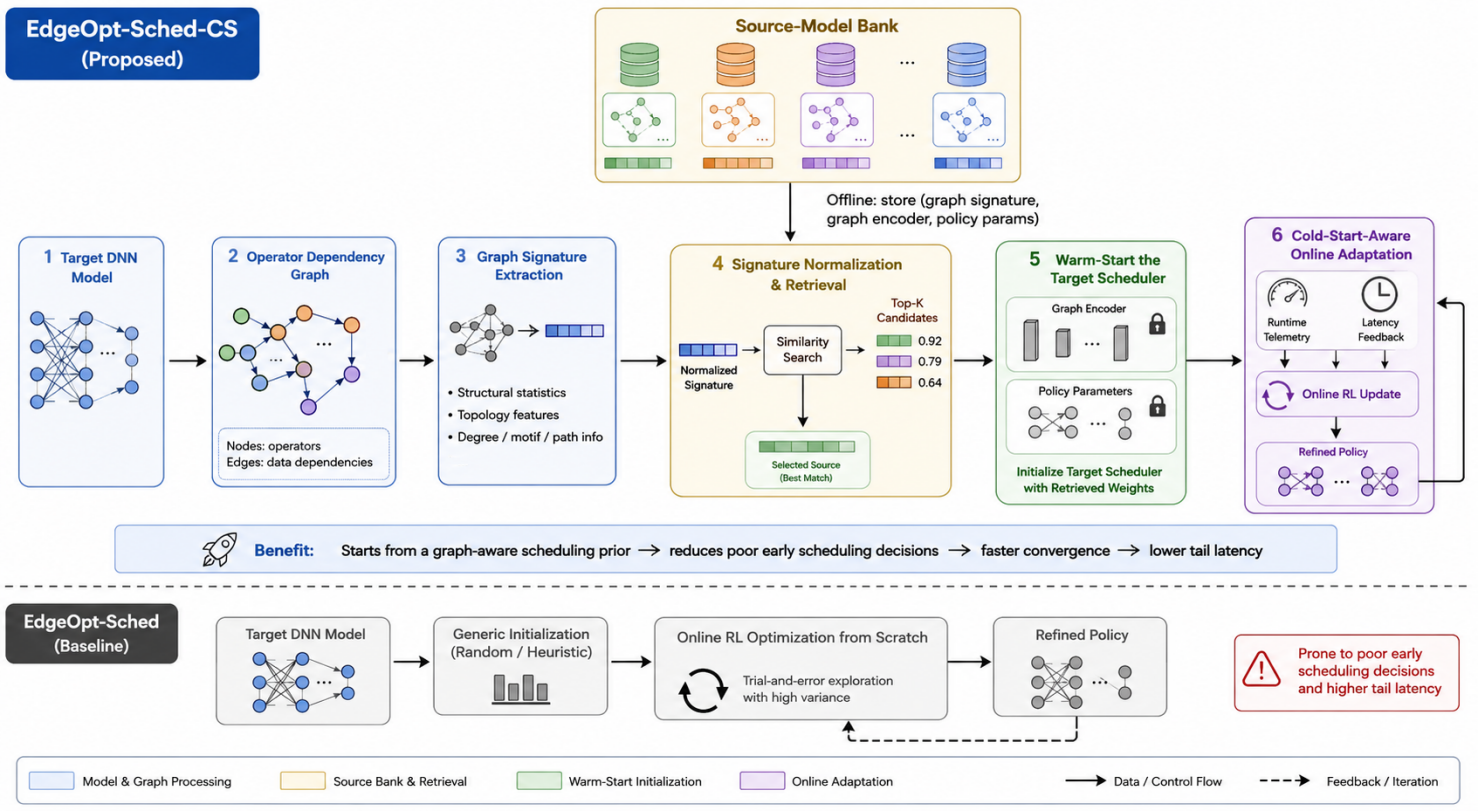
Overview of EdgeOpt-Sched-CS. A newly deployed DNN is converted into an operator dependency graph, from which a compact graph signature is extracted. The signature is used to retrieve a structurally relevant source scheduler from the source-model bank, and the retrieved encoder and policy parameters warm-start the target scheduler. During the cold-start window, online adaptation further refines scheduling decisions using runtime telemetry and latency feedback. This graph-aware initialization reduces poor early scheduling decisions compared with the generic initialization used by the original EdgeOpt-Sched.

Figure 1 illustrates the overall architecture of EdgeOpt-Sched-CS. Compared with the original EdgeOpt-Sched pipeline, which initializes the scheduler from a generic state and relies on online adaptation after deployment, EdgeOpt-Sched-CS retrieves a structurally relevant source scheduler from the source-model bank and uses it to warm-start the target scheduler. As a result, the target policy begins from a more informative region of the parameter space, reducing poor early scheduling decisions during the cold-start window.

### 3.2. Problem Setting and Cold-Start Objective

Let G=(V,E) denote the operator dependency graph of a target DNN model, where *V* is the set of operators and *E* is the set of tensor dependencies. Following the original EdgeOpt-Sched formulation, each operator $v_{i}\in V$ is associated with execution-related attributes such as operator type, tensor shape, compute intensity, memory footprint, and fusion compatibility. At each scheduling step *t*, the scheduler observes a state

(1) $$s_{t}=\mathrm{concat}\left(h_{i},S_{t}\right),$$

where $h_{i}$ is the GNN embedding of a ready operator and $S_{t}$ is the runtime telemetry vector, including memory availability, device load, temperature, and partial execution statistics. The scheduler then chooses an action $a_{t}$, corresponding to the next executable operator or a local transformation such as reordering, fusion, or buffer reuse.

The original objective focuses on maximizing long-term cumulative reward:

(2) $$\max_{\pi }E\left[\sum_{t=1}^{T}r_{t}\right],$$

where $r_{t}$ reflects latency, peak memory, latency variance, and switching cost. While suitable for steady-state optimization, this formulation does not explicitly distinguish the cold-start phase from the later stabilized phase.

To make this distinction explicit, we introduce a *cold-start window* of length $T_{c}$, covering the early inference steps immediately after model loading. Let $L_{t}^{\pi }$ denote the end-to-end latency achieved at step *t* under policy π, and let $L_{ss}$ denote the stabilized latency achieved after convergence. We define the cold-start regret as

(3) $$R_{cs}\left(\pi \right)=\sum_{t=1}^{T_{c}}\left(L_{t}^{\pi }-L_{ss}\right).$$

This term measures how much extra latency is paid during early deployment before the scheduler reaches stable behavior.

Our optimization goal is therefore revised as

(4) $$\max_{\pi }E\left[\sum_{t=1}^{T}r_{t}\right]-\omega R_{cs}\left(\pi \right),$$

where ω controls the importance of deployment-time efficiency relative to the original long-term reward.

### 3.3. Model Signature Construction

The three groups of signature features are designed to capture complementary factors that influence operator scheduling. Topology-level features determine the amount of scheduling freedom, dependency depth, and critical-path constraints. For example, graphs with long critical paths offer fewer opportunities for reordering, whereas graphs with larger ready sets provide more scheduling alternatives. Operator-composition features describe the mixture of compute-intensive, memory-intensive, layout-transforming, and fusible operators, which directly affects whether a scheduling policy learned on one graph can be reused on another. Cost-level features summarize coarse latency and memory behavior and are therefore particularly relevant to early scheduling decisions on resource-constrained devices. Although these descriptors are manually defined, they are lightweight, interpretable, and available before full online adaptation, making them suitable for deployment-time retrieval.

A central question in cold-start optimization is how to determine whether previously learned scheduling knowledge is relevant to a newly deployed target graph. To answer this, we construct for each model a compact *model signature* that summarizes its structural and semantic properties in a way that is predictive of scheduling behavior.

Given a graph *G*, we define its signature as

(5) $$z\left(G\right)=\left[z_{topo};z_{op};z_{cost}\right],$$

where $z_{topo}$, $z_{op}$, and $z_{cost}$ denote topology-level, operator-level, and cost-level descriptors, respectively.

#### 3.3.1. Topology-Level Features

The topology component captures the graph organization that shapes scheduling freedom and dependency constraints:

(6) $$z_{topo}=\left[|V|,\;|E|,\;d_{in}^{avg},\;d_{out}^{avg},\;l_{cp},\;b_{avg},\;r_{avg}\right],$$

where |V| and |E| are the numbers of nodes and edges, $d_{in}^{avg}$ and $d_{out}^{avg}$ are the average in-/out-degree, $l_{cp}$ is the critical-path length, $b_{avg}$ is the average branching factor, and $r_{avg}$ is the average size of the ready set during topological simulation.

#### 3.3.2. Operator-Level Features

The operator component describes the semantic composition of the graph:

(7) $$z_{op}=\left[\rho_{conv},\;\rho_{matmul},\;\rho_{reshape},\;\rho_{transpose},\;\rho_{eltwise},\;\rho_{lat},\;\rho_{mem},\;\rho_{fus}\right],$$

where each ρ denotes the fraction of a given operator type or class, such as convolution, MatMul, layout-transforming operators, element-wise operators, latency-bound operators, memory-bound operators, and fusible operators.

#### 3.3.3. Cost-Level Features

The cost component summarizes coarse runtime characteristics obtained through a short profiling pass. Let $c_{i}$, $m_{i}$, and $p_{i}$ denote the estimated compute cost, memory demand, and parallelizability of operator *i*. We then compute

(8) $$z_{cost}=\left[\bar{c},\;\bar{m},\;\sigma_{c},\;\sigma_{m},\;m_{peak}^{est},\;\rho_{crit}\right],$$

where $\bar{c}$ and $\bar{m}$ are the average compute and memory estimates, $\sigma_{c}$ and $\sigma_{m}$ are their standard deviations, $m_{peak}^{est}$ is the estimated peak memory, and $\rho_{crit}$ is the fraction of nodes on the critical path.

Because the raw signature components have heterogeneous scales, directly computing distances over z(G) would cause large-magnitude features, such as |V| or |E|, to dominate bounded-ratio features such as $\rho_{conv}$ or $\rho_{fus}$. To avoid this issue, we normalize each signature dimension before retrieval. Specifically, for the *j*-th feature dimension, we compute

(9) $${\tilde{z}}_{j}\left(G\right)=\frac{z_{j}\left(G\right)-\mu_{j}}{\sigma_{j}+\varepsilon },$$

where $\mu_{j}$ and $\sigma_{j}$ are the mean and standard deviation of the *j*-th feature estimated over the source-model bank, and ε is a small constant for numerical stability. The normalized signature is then written as

(10) $$\tilde{z}\left(G\right)=\left[{\tilde{z}}_{topo};{\tilde{z}}_{op};{\tilde{z}}_{cost}\right].$$

Furthermore, not all feature groups contribute equally to scheduling quality. In particular, cost-related features are generally more informative for operator scheduling than raw graph size alone. Therefore, we introduce a non-negative weight vector

(11) $$w=\left[w_{1},w_{2},\ldots ,w_{d}\right],\;w_{j}\geq 0,$$

or equivalently a diagonal weighting matrix W=diag(w), to modulate the influence of different signature dimensions during retrieval. Empirically, higher weights are assigned to cost-sensitive and operator-composition features than to coarse topology-count features, with the optimal weight configuration determined via a grid search on the source-model bank.

The weighting matrix is introduced to emphasize scheduling-relevant dimensions rather than to create a complex retrieval model. In practice, raw topology counts such as |V| and |E| can have much larger numerical variation than normalized operator ratios. Without weighting, retrieval may be dominated by graph size rather than by scheduling behavior. Therefore, we assign larger weights to cost-sensitive and operator-composition features and tune the weights on the source-model bank. This design keeps retrieval inexpensive and interpretable while allowing the distance metric to better reflect scheduling similarity.

### 3.4. Graph-Signature-Based Retrieval and Warm-Start Initialization

Once a target signature is computed, the scheduler searches a source-model bank

(12) $$B={\left\{(G_{k},\tilde{z}\left(G_{k}\right),\theta_{k},\phi_{k})\right\}}_{k=1}^{K},$$

where each entry contains a previously seen graph, its normalized signature, the trained policy parameters $\theta_{k}$, and encoder parameters $\phi_{k}$.

The target graph retrieves the most relevant source graph according to a weighted distance over normalized signatures:

(13) $$G^{★}=\arg\min_{G_{k}\in B}{∥W\left(\tilde{z}\left(G_{t}\right)-\tilde{z}\left(G_{k}\right)\right)∥}_{2}.$$

Here, $\tilde{z}\left(G\right)$ denotes the normalized model signature and W is a diagonal weighting matrix that adjusts the importance of different feature dimensions. This formulation prevents large-scale topology features from dominating the retrieval score and allows the retrieval process to better reflect scheduling-relevant structural and cost similarities.

After retrieval, EdgeOpt-Sched-CS initializes the target scheduler using the retrieved source parameters:

(14) $$\theta_{t}^{\left(0\right)}\leftarrow \theta^{★},\;\phi_{t}^{\left(0\right)}\leftarrow \phi^{★}.$$

Here, $\theta_{t}^{\left(0\right)}$ denotes the initial policy parameters for the target graph and $\phi_{t}^{\left(0\right)}$ denotes the initial GNN encoder parameters. Unlike the original EdgeOpt-Sched pipeline, which begins adaptation from a generic pre-trained state and refines it through warm-up updates, the proposed method begins from a graph-aware prior.

In addition to nearest-neighbor transfer, the same framework naturally supports a meta-initialized scheduler. In that setting, instead of retrieving only one source model, the source-model bank is used to learn a shared initialization $\left(\theta_{\mathrm{meta}},\phi_{\mathrm{meta}}\right)$ that can be rapidly adapted to many target graphs.

We use an interpretable signature-based retrieval method rather than a learned retrieval model for two reasons. First, the source-model bank is modest in size, and a learned similarity model may overfit when only a limited number of source graphs are available. Second, deployment-time retrieval must remain lightweight enough to avoid becoming a new cold-start bottleneck. Nevertheless, learning a graph-similarity metric from a larger and more diverse source-model bank is a promising extension and is discussed as future work.

### 3.5. Cold-Start-Aware Online Adaptation

Warm-start initialization alone is insufficient because even similar graphs are not identical. The target scheduler must still adapt to the actual hardware state, device telemetry, and exact tensor shapes observed during deployment. Therefore, EdgeOpt-Sched-CS retains the online adaptation loop of the original scheduler but modifies its early-stage learning behavior to explicitly prioritize cold-start performance.

Let $T_{c}$ denote the cold-start window. During the first $T_{c}$ inference steps, we use a time-varying reward

(15) $$r_{t}^{cs}=r_{t}-\lambda_{t}L_{t},$$

where $r_{t}$ is the original latency–memory reward and $\lambda_{t}$ is a decaying coefficient defined as

(16) $$\lambda_{t}=\lambda_{0}\exp\left(-t/\tau \right),\;1\leq t\leq T_{c}.$$

This design assigns greater weight to latency in the earliest steps and gradually reduces that extra emphasis as the scheduler approaches stable operation.

A potential concern is that a large early-stage latency penalty may bias the scheduler toward overly myopic decisions and thus trap the policy in a poor local optimum. We mitigate this risk in three ways. First, the penalty coefficient $\lambda_{t}$ decays exponentially and only affects the early deployment window, so its influence vanishes as the scheduler approaches steady-state optimization. Second, the policy update is still governed by PPO, whose clipped surrogate objective limits excessively large policy shifts between consecutive updates:

(17) $$L_{\mathrm{PPO}}^{\mathrm{clip}}=E_{t}\left[\min\left(r_{t}\left(\theta \right){\hat{A}}_{t},\;\mathrm{clip}\;\left(r_{t}\left(\theta \right),1-\epsilon ,1+\epsilon \right){\hat{A}}_{t}\right)\right],$$

where $r_{t}\left(\theta \right)$ is the probability ratio and ${\hat{A}}_{t}$ is the estimated advantage. This clipping mechanism constrains aggressive updates even when the early-stage reward shaping is relatively strong. Third, warm-start initialization already places the target scheduler in a more favorable region of the policy space, thereby reducing the likelihood that the cold-start penalty alone drives learning toward a poor solution. In this sense, the penalty term acts as a transient guidance signal rather than a permanent distortion of the long-term optimization objective.

### 3.6. Training Objective

To support transfer across models, we train the source-model bank using a two-stage procedure. In the first stage, source schedulers are trained on a diverse collection of representative CNN and transformer graphs. In the second stage, cold-start-aware transfer episodes are constructed by retrieving one or more source schedulers and running short adaptation episodes on target graphs.

The optimization objective becomes

(18) $$L=L_{ppo}+\alpha L_{cs}+\beta L_{reg},$$

where $L_{ppo}$ is the standard PPO loss, $L_{cs}$ is a cold-start penalty term derived from the cumulative latency within the first $T_{c}$ steps, and $L_{reg}$ is an optional regularizer that prevents unstable drift from the transferred prior.

One practical choice is

(19) $$L_{cs}=\sum_{t=1}^{T_{c}}w_{t}L_{t},$$

where $w_{t}$ is a monotonically decreasing weight sequence that emphasizes the earliest requests.

### 3.7. Computational Complexity and Overhead

An important design requirement is that the proposed cold-start optimization should not introduce a heavy new bottleneck. The additional cost of EdgeOpt-Sched-CS consists of three parts: signature extraction, source retrieval, and optional transfer regularization.

Signature extraction is linear in graph size:

(20) O(|V|+|E|),

because it mainly traverses the ODG and aggregates per-node statistics. Retrieval over a source-model bank of size *K* with signature dimension *d* incurs

(21) O(Kd),

which is negligible when *K* is modest.

### 3.8. Algorithm Description

Algorithm 1 summarizes the overall workflow of EdgeOpt-Sched-CS.
**Algorithm 1:** EdgeOpt-Sched-CS: Cold-Start-Aware Dynamic Scheduling
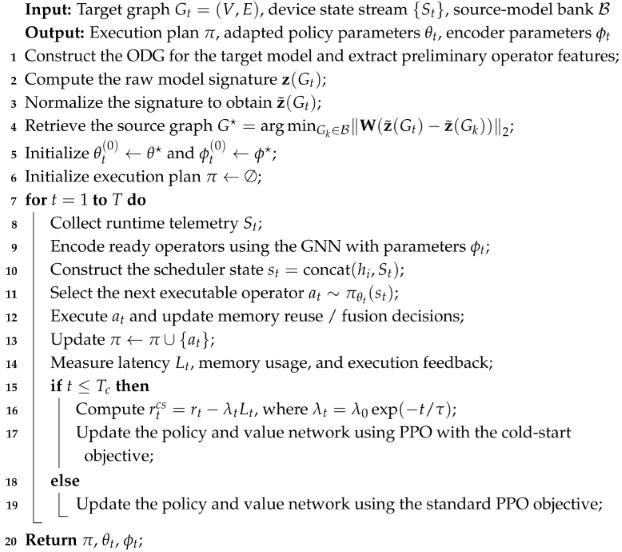


## 4. Experimental Design

To rigorously evaluate the effectiveness of EdgeOpt-Sched-CS, we design a cold-start-oriented experimental protocol that measures both deployment-time efficiency and steady-state scheduling quality. In contrast to conventional evaluations that focus primarily on average latency or throughput after convergence, our experiments explicitly separate the *cold-start phase* from the *steady-state phase*. This design allows us to quantify whether the proposed warm-start mechanism reduces early-stage overhead while preserving the long-term advantages of dynamic graph scheduling.

### 4.1. Hardware Platforms and Software Environment

We conduct experiments on three representative edge platforms:

- **Raspberry Pi 4**: ARM Cortex-A72 (4 cores, 1.5 GHz), 4 GB RAM; (The Raspberry Pi Foundation, Cambridge, UK)
- **Raspberry Pi 5**: ARM Cortex-A76 (4 cores, 2.4 GHz), 8 GB RAM; (The Raspberry Pi Foundation, Cambridge, UK)
- **Orange Pi 5**: ARM Cortex-A76 (4 cores, 2.4 GHz), 16 GB RAM. (The Raspberry Pi Foundation, Cambridge, UK)

All experiments are conducted under Ubuntu 22.04. The software stack includes ONNX Runtime, TVM, and our customized EdgeOpt-Sched-CS framework.

The selected device–model scenarios are intended to cover representative edge-inference settings with different resource and workload characteristics. Raspberry Pi 4 represents low-cost and resource-constrained sensing nodes commonly used in lightweight vision analytics, intelligent monitoring, and industrial Internet of Things deployments. Raspberry Pi 5 represents a stronger edge gateway class, where moderate transformer inference and multi-model workloads are increasingly feasible. Orange Pi 5 provides a memory-richer edge platform and is used to evaluate larger transformer-style and quantized language-model workloads. On the model side, MobileNetV3 represents lightweight convolutional neural network inference for mobile vision; BERT represents medium-scale transformer inference for language understanding, embedding, and classification tasks; while DeepSeek-1B and quantized Qwen2-7B represent structurally complex edge large-language-model workloads. This selection allows us to study cold-start behavior across both simple CNN-style graphs and deeper, more heterogeneous transformer-style graphs.

### 4.2. Workloads and Source–Target Split

A key objective of this work is to evaluate whether prior scheduling knowledge can be transferred from previously seen graphs to unseen target graphs. To this end, we divide the workloads into a *source-model bank* and a *target-model set*.

#### 4.2.1. Source-Model Bank

The source-model bank contains representative models covering both convolutional and transformer-style architectures, including Bidirectional Encoder Representations from Transformers (BERT), Vision Transformer (ViT), and Residual Network (ResNet) families:

- BERT-base;
- BERT-large;
- ViT-B;
- ViT-L;
- ResNet-50;
- ResNet-101.

#### 4.2.2. Target-Model Set

The target-model set is used for cold-start evaluation. We consider both representative deployment targets and additional unseen graphs for generalization analysis:

- MobileNetV3;
- BERT deployment variant, used to evaluate transfer to a related but separately deployed transformer graph;
- DeepSeek-1B;
- Qwen2-7B (quantized);
- Unseen target graphs including EfficientNet-B0, ShuffleNetV2, DistilBERT, TinyBERT, DeiT-Tiny, and MiniViT.

### 4.3. Baselines

We compare EdgeOpt-Sched-CS with three groups of baselines: static runtime/compiler baselines, an online dynamic scheduling baseline, and transfer-oriented warm-start baselines. This grouping allows us to evaluate not only whether the proposed method outperforms static execution and online-only scheduling but also whether graph-signature-based retrieval is more effective than simpler warm-start strategies.

#### 4.3.1. Static Runtime and Compiler Baselines

- **ONNX Runtime**: a widely used inference runtime based on the Open Neural Network Exchange representation.
- **TVM**: a compiler-based deployment baseline using graph lowering and backend-specific optimization.

#### 4.3.2. Dynamic Scheduling Baseline

- **Original EdgeOpt-Sched**: the online dynamic scheduler without cold-start-aware graph-signature retrieval or warm-start initialization.

#### 4.3.3. Transfer-Oriented Baselines and Variants

- **Random source transfer**: a source scheduler is selected uniformly at random from the source-model bank.
- **Worst-match transfer**: the source scheduler with the largest graph-signature distance is intentionally selected to evaluate robustness against mismatched priors.
- **Encoder-only transfer**: only the GNN encoder parameters are transferred, while the policy is initialized generically.
- **Policy-only transfer**: only the scheduling policy parameters are transferred, while the graph encoder is initialized generically.
- **Transfer-Init**: both encoder and policy parameters are transferred using graph-signature retrieval, but without the full cold-start-aware reward shaping.
- **Meta-Init**: a shared initialization learned from the source-model bank is used instead of target-specific nearest-source retrieval.
- **EdgeOpt-Sched-CS**: the full proposed method with graph-signature retrieval, encoder–policy warm-start initialization, and cold-start-aware online adaptation.

We note that several recent edge scheduling systems focus on request-level dispatching, edge–cloud partitioning, or pipeline-level workload allocation. These systems are highly relevant from a deployment perspective, but they optimize a different scheduling granularity from the operator-level graph scheduling problem studied in this paper. Therefore, they are discussed in Section 2, while the experimental comparison focuses on methods that can be evaluated under the same operator-level deployment protocol.

Including such systems as direct baselines would require changing the problem setting from intra-graph operator scheduling to request-level serving or edge–cloud orchestration, which would make the comparison less controlled.

### 4.4. Evaluation Metrics

To fully characterize deployment behavior, we report both *cold-start metrics* and *steady-state metrics*.

#### 4.4.1. Cold-Start Metrics

First-Ready Time (FRT).

First-Ready Time is defined as the elapsed time from model loading to the first point at which the scheduler becomes operational.

Cumulative Latency@N.

For the first *N* inference requests, we compute

(22) $$\mathrm{CL}@N=\sum_{t=1}^{N}L_{t}.$$

Time-to-Stability (TTS).

Let ${\bar{L}}_{t-w+1:t}$ denote the moving-average latency over a window of size *w*. Time-to-Stability is defined as:

(23) $$\mathrm{TTS}=\min\left\{t\geq w\;|\;{\bar{L}}_{t-w+1:t}\leq 1.05\;L_{ss}\right\}.$$

Cold-Start Regret.

(24) $$R_{cs}=\sum_{t=1}^{T_{c}}\left(L_{t}-L_{ss}\right).$$

#### 4.4.2. Steady-State Metrics

Steady-State Latency.

(25) $$L_{ss}=\frac{1}{T_{s}}\sum_{t=T_{c}+1}^{T_{c}+T_{s}}L_{t}.$$

Peak Memory Usage.

Peak memory usage is measured as the maximum runtime memory footprint observed during inference execution.

Scheduler Overhead.

Scheduler overhead is defined as the time consumed by graph encoding, action selection, and scheduler-side update logic, excluding actual operator execution time.

Latency Variance.

We additionally report the variance of per-step latency within both the cold-start and steady-state windows.

### 4.5. Experimental Protocol

The 50-step deployment episode is designed to capture both the initial adaptation behavior and the stabilized execution phase in a controlled and repeatable manner. The first 20 inference steps are treated as the cold-start window because the original dynamic scheduler exhibits the largest latency variation and policy adaptation during this interval in our preliminary measurements. Steps 21–50 are then used to estimate steady-state behavior after the most volatile adaptation period. This protocol is not intended to model all long-term serving conditions but to isolate the effect of scheduler initialization on deployment-onset efficiency.

For every combination of device, model, and method, we apply the same cold-start deployment procedure to ensure that the observed differences primarily reflect scheduler initialization and adaptation behavior rather than differences in measurement setup. Each experiment is performed under a full cold-start deployment setting. For every combination of device, model, and method, we reset the runtime state and execute the following procedure:

1. Reset the scheduler state and clear software-side runtime caches relevant to scheduling;
2. Apply the same fixed pre-measurement procedure to all methods in order to reduce the influence of CPU frequency ramp-up, cache warming, and file I/O initialization effects as much as possible;
3. Load the target model and construct its operator dependency graph;
4. For EdgeOpt-Sched-CS variants, compute the model signature and perform source retrieval/warm-start initialization;
5. Execute 50 consecutive inference steps under the same input configuration;
6. Record steps 1–20 as the *cold-start window*;
7. Record steps 21–50 as the *steady-state window*;
8. Log per-step latency, scheduler overhead, peak memory, CPU utilization, and device temperature.

We repeat each configuration 10 times using different random seeds and report average results across runs.

### 4.6. Ablation Settings

Among the transfer-oriented baselines listed above, the following configurations are also used as ablation settings to isolate the contribution of retrieval quality, encoder transfer, policy transfer, and cold-start-aware reward shaping:

- **Without retrieval**: the scheduler uses a generic initialization instead of graph-signature-based source selection;
- **Policy-only transfer**: only the policy network is transferred, while the graph encoder is initialized generically;
- **Encoder-only transfer**: only the graph encoder is transferred;
- **Without cold-start-aware reward**: warm-start initialization is retained but the early-stage latency penalty is removed;
- **Random source transfer**: a source model is sampled uniformly from the source-model bank instead of being selected by signature similarity;
- **Worst-match transfer**: the target graph is intentionally initialized from the least similar source graph in the source-model bank, in order to evaluate robustness against severely mismatched priors.

### 4.7. Statistical Analysis

All reported results are presented as mean ± standard deviation over 10 independent runs. For pairwise comparisons between the original EdgeOpt-Sched and the proposed cold-start-aware variants, we use paired *t*-tests when the normality assumption is satisfied. When normality is violated, we additionally report Wilcoxon signed-rank tests. For multiple comparisons, adjusted *p*-values are computed using the Holm–Bonferroni correction. We quantify practical significance using Cohen’s *d*.

### 4.8. Implementation Details

Unless otherwise specified, the GNN encoder uses a 2-layer architecture with hidden size 128, and the policy network is implemented as a lightweight multilayer perceptron. During target deployment, retrieval is performed using a weighted normalized Euclidean distance over the signature vector, where each signature dimension is first standardized with bank-level statistics and then modulated by a diagonal weighting matrix W. In practice, larger weights are assigned to cost-sensitive and operator-composition features than to coarse topology-count features. The PPO update frequency, learning rate, and replay configuration follow the same default settings as the original EdgeOpt-Sched framework to isolate the effect of the proposed cold-start optimization.

### 4.9. Threats to Validity

We note several factors that may affect the generality of the reported results. First, the quality of warm-start initialization depends on the diversity of the source-model bank. Second, although our evaluation covers multiple edge devices, hardware diversity in real deployment can be broader. Third, the present study uses a controlled cold-start deployment protocol for consistent comparison across methods; broader practical value should ultimately be confirmed under long-horizon real serving traces.

In addition, real edge deployments may involve bursty request arrivals, background workload contention, thermal drift, and changing input distributions. The controlled 50-step protocol does not fully reproduce all these factors. Instead, it provides a repeatable setting for isolating and comparing cold-start scheduler behavior. We therefore interpret the results as evidence of reduced deployment-onset overhead under controlled cold-start conditions, while long-horizon serving with dynamic workload arrivals remains an important direction for future evaluation.

## 5. Results

In this section, we evaluate EdgeOpt-Sched-CS from six perspectives. We first examine whether the proposed method reduces deployment-time latency during the cold-start phase. We then analyze whether it shortens the time required to reach stable performance, whether it preserves the steady-state latency–memory behavior of the original dynamic scheduler, and whether it generalizes to unseen target graphs. Finally, we study the contribution of graph-signature-based retrieval and quantify the additional overhead introduced by the cold-start-aware design.

### 5.1. RQ1: Does EdgeOpt-Sched-CS Reduce Cold-Start Latency

**Figure 2:**
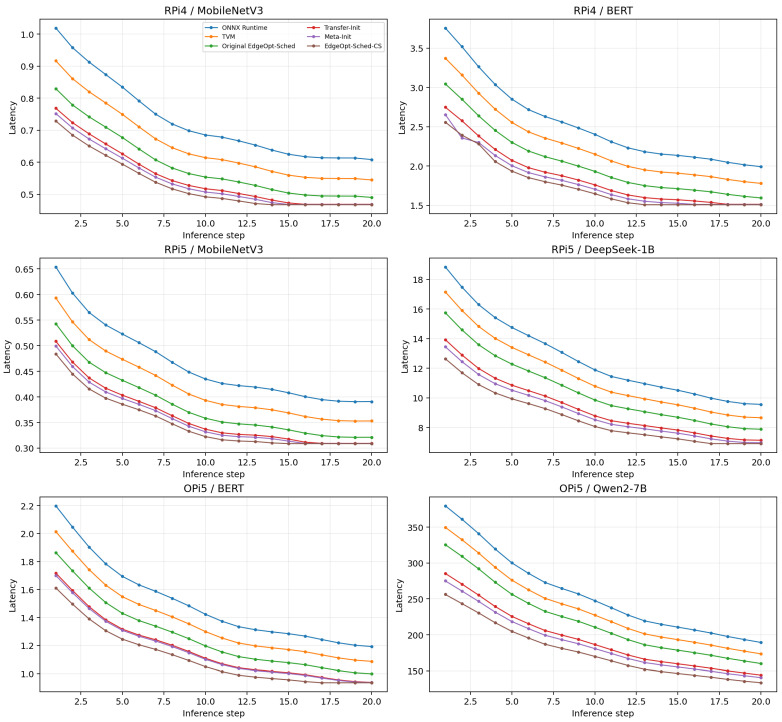
Cold-start latency curves across six deployment scenarios. Each subfigure shows the first 20 inference steps under cold-start deployment. EdgeOpt-Sched-CS reduces early-stage latency more rapidly than the original EdgeOpt-Sched and exhibits more stable convergence behavior across heterogeneous edge workloads.

We first evaluate the latency trajectory during the initial deployment phase. Figure 2 presents a grouped comparison of cold-start latency curves across six representative deployment scenarios, including lightweight CNN workloads, medium-scale transformer inference, and large quantized language models on heterogeneous edge platforms. Compared with the original EdgeOpt-Sched, both warm-start variants reduce latency during early inference, while the full EdgeOpt-Sched-CS consistently achieves the lowest latency trajectory in most scenarios.

The magnitude of improvement is clearly scenario-dependent. According to Table 1, the reduction in CL@10 is 11.9% on RPi4/MobileNetV3, 15.3% on RPi4/BERT, 10.6% on RPi5/MobileNetV3, 19.1% on RPi5/DeepSeek-1B, 13.0% on OPi5/BERT, and 20.4% on OPi5/Qwen2-7B. In absolute terms, the corresponding CL@10 values decrease from 6.69 to 5.90, from 23.60 to 19.99, from 4.33 to 3.87, from 123.27 to 99.75, from 14.60 to 12.70, and from 2588.03 to 2061.03, respectively. This non-uniform pattern is more consistent with realistic deployment behavior than a fixed proportional improvement across all tasks. It suggests that warm-start transfer is most valuable when the target graph has deeper dependencies, more heterogeneous operators, and a larger space of potentially poor early execution orders.

**Figure 3:**
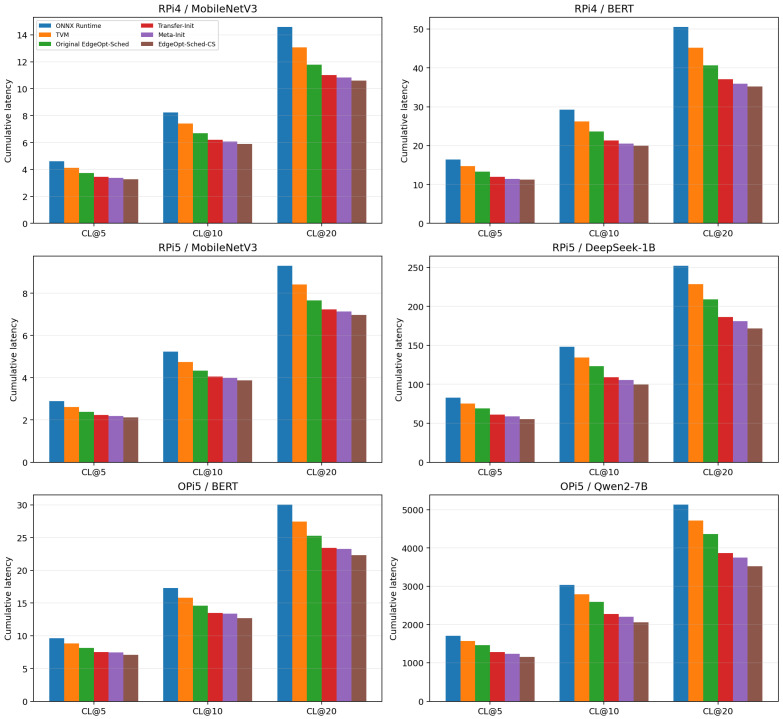
Cumulative latency comparisons across six deployment scenarios. Each subfigure reports CL@5, CL@10, and CL@20 for six runtime or scheduling variants. EdgeOpt-Sched-CS consistently yields the lowest cumulative latency across the cold-start window.

To quantify the cumulative effect of this improvement, Figure 3 compares CL@5, CL@10, and CL@20 across the same six deployment scenarios. Across all tested platforms, EdgeOpt-Sched-CS achieves lower cumulative latency than the original dynamic scheduler, indicating that the benefit is not limited to isolated steps but persists throughout the full cold-start window.

Overall, the cumulative-latency results show that EdgeOpt-Sched-CS reduces deployment-time cost in a consistent but non-uniform manner. The reduction in CL@10 ranges from 10.6% to 20.4%, with an average improvement of approximately 15.1% across the six representative deployments.

### 5.2. RQ2: Does EdgeOpt-Sched-CS Shorten Time-to-Stability?

We next study how quickly each method approaches stable performance after deployment. Following Section 4, Time-to-Stability (TTS) is defined as the earliest inference step at which the windowed moving-average latency falls within 5% of the steady-state latency.

**Figure 4:**
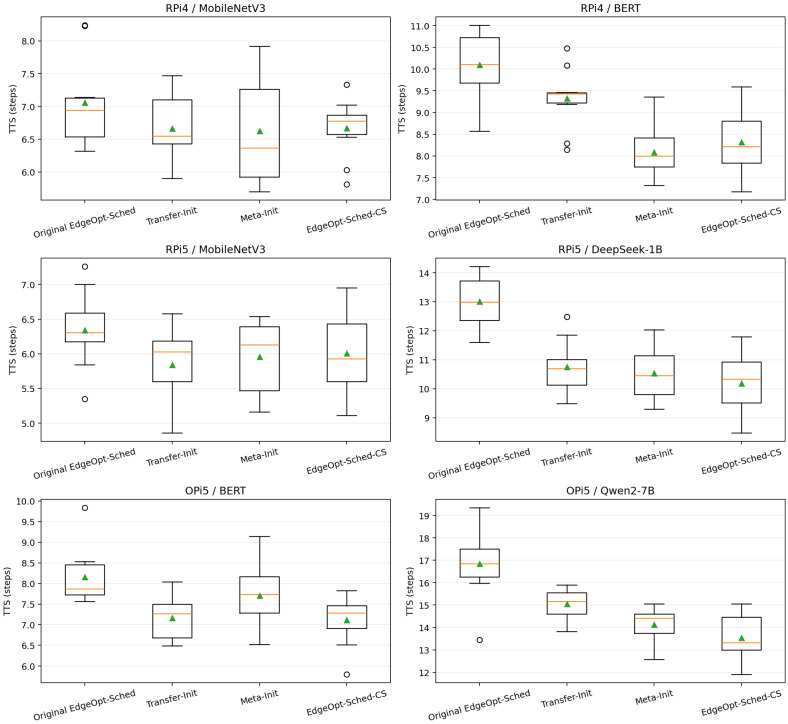
Time-to-Stability (TTS) distributions across six deployment scenarios. Each subfigure summarizes repeated runs under cold-start deployment. EdgeOpt-Sched-CS reaches stable performance in fewer inference steps than the original EdgeOpt-Sched, with the strongest reductions observed on larger transformer-style workloads.

Figure 4 shows the distribution of TTS across repeated runs for six representative device–model combinations. EdgeOpt-Sched-CS consistently shortens the adaptation horizon relative to the original EdgeOpt-Sched. However, the reduction is again heterogeneous across scenarios. The mean TTS decreases from 7.06±0.69 to 6.67±0.45 on RPi4/MobileNetV3, from 10.10±0.75 to 8.33±0.81 on RPi4/BERT, from 6.34±0.55 to 6.01±0.60 on RPi5/MobileNetV3, from 13.01±0.88 to 10.19±1.08 on RPi5/DeepSeek-1B, from 8.16±0.69 to 7.11±0.60 on OPi5/BERT, and from 16.85±1.59 to 13.53±1.12 on OPi5/Qwen2-7B.

In relative terms, the TTS reduction is 5.5%, 17.6%, 5.2%, 21.7%, 12.8%, and 19.7% for the six scenarios, respectively. The two MobileNetV3 deployments exhibit only modest TTS gains, indicating that the base dynamic scheduler already stabilizes quickly on lightweight workloads. By contrast, the improvement becomes much stronger on structurally complex transformer-style workloads, especially DeepSeek-1B and Qwen2-7B.

This pattern further supports the intuition that cold-start-aware initialization is most beneficial when the deployment graph is structurally complex and the baseline warm-up horizon is long. In simpler workloads, the method still helps, but the main benefit comes from reducing early cumulative latency rather than drastically shortening convergence time.

Taken together with the cumulative-latency results, the TTS analysis indicates that the proposed cold-start-aware initialization reduces both *how much* latency is paid during deployment onset and *how long* the system remains in a suboptimal adaptation state. Averaged over all six representative scenarios, the TTS reduction is approximately 13.8%, while the actual per-scenario range spans from 5.2% to 21.7%.

### 5.3. RQ3: Does EdgeOpt-Sched-CS Preserve Steady-State Performance

A critical question is whether improving the cold-start phase comes at the expense of long-term execution quality. To answer this, we compare steady-state latency, peak memory usage, and scheduler overhead after the cold-start window has ended.

It should be emphasized that EdgeOpt-Sched-CS is primarily designed to reduce cold-start deployment overhead rather than to substantially improve the final steady-state scheduler. Therefore, the steady-state results are interpreted conservatively. The key question in this subsection is whether the proposed cold-start mechanism preserves the latency–memory behavior of the original dynamic scheduler after convergence.

**Figure 5:**
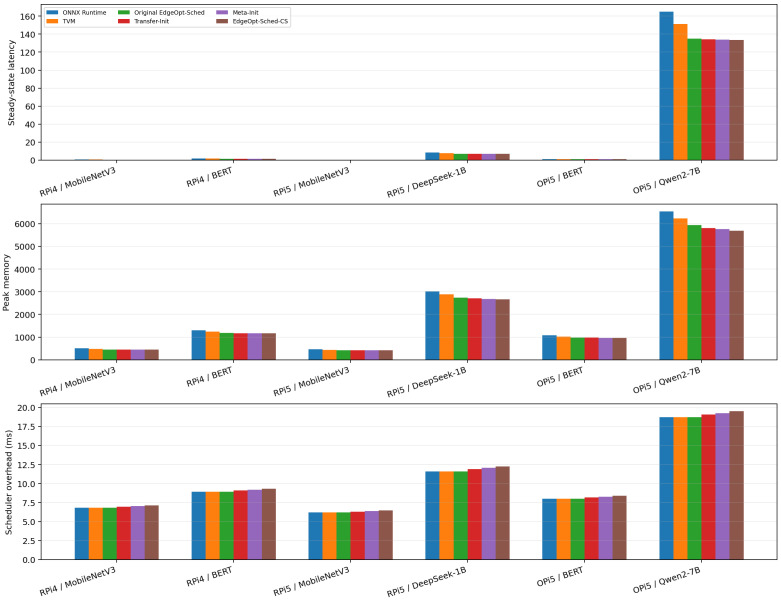
Steady-state comparisons across deployment scenarios. From top to bottom, the grouped charts report steady-state latency, peak memory usage, and scheduler overhead. EdgeOpt-Sched-CS maintains comparable steady-state latency–memory behavior while adding only a small overhead increase.

Figure 5 reports steady-state comparisons across all six deployment scenarios. Several observations can be made. First, EdgeOpt-Sched-CS preserves the steady-state latency behavior of the original EdgeOpt-Sched and shows only small numerical differences after convergence. Specifically, steady-state latency decreases from 0.470 to 0.467, from 1.520 to 1.509, from 0.310 to 0.309, from 6.980 to 6.901, from 0.940 to 0.935, and from 135.100 to 133.411 across the six scenarios. These correspond to relative reductions ranging from about 0.3% to 1.3%.

Second, peak memory usage is also reduced, but with a wider spread that is more plausible for heterogeneous models. The observed reductions are from 460.0 to 457.1 MB, from 1180.0 to 1163.8 MB, from 420.0 to 418.3 MB, from 2746.0 to 2667.4 MB, from 980.0 to 968.0 MB, and from 5938.0 to 5689.8 MB. In relative terms, this corresponds to reductions ranging from about 0.4% to 4.2%.

Third, scheduler overhead increases slightly in all scenarios. The overhead rises from 6.80 to 7.11 ms, from 8.90 to 9.33 ms, from 6.20 to 6.45 ms, from 11.60 to 12.26 ms, from 8.00 to 8.39 ms, and from 18.70 to 19.49 ms. The absolute increase therefore ranges from 0.25 ms to 0.79 ms per decision.

These results suggest that the proposed cold-start optimization is complementary to the original dynamic scheduling objective. Rather than trading steady-state quality for faster startup, EdgeOpt-Sched-CS improves the early phase while keeping the steady-state latency–memory balance essentially unchanged. Importantly, the slight increase in scheduler overhead is far smaller than the deployment-time savings obtained during the cold-start window.

Overall, the steady-state improvements are modest and should not be viewed as the main contribution of the method. The more important observation is that cold-start-aware initialization reduces early deployment latency without degrading the steady-state latency–memory behavior of the original EdgeOpt-Sched framework.

### 5.4. RQ4: Does the Proposed Method Generalize to Unseen Target Graphs

We next examine whether the proposed transfer mechanism generalizes to target graphs that are not directly optimized as source schedulers. This question is important because the method is intended for realistic deployment, where newly introduced models may differ from those used to construct the source-model bank.

**Figure 6:**
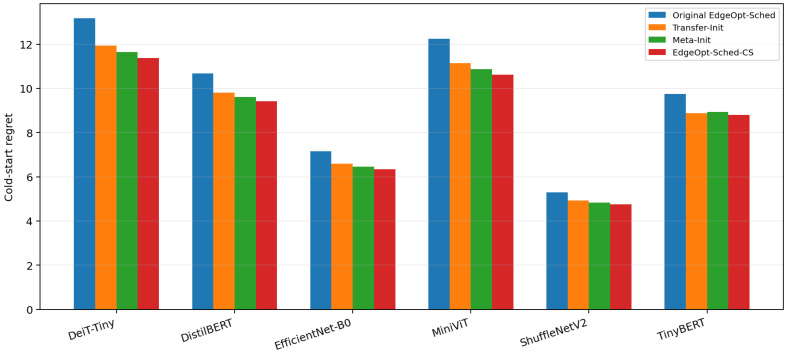
Generalization to unseen target graphs. The chart compares cold-start regret on six unseen models. EdgeOpt-Sched-CS consistently outperforms the original online-only dynamic scheduler, indicating that the learned scheduling prior transfers across heterogeneous graph structures.

Figure 6 compares cold-start regret on multiple unseen target models spanning CNN, compact language-model, and lightweight vision-transformer families. The results show that graph-signature-based warm-start remains effective even when the target graph has not been explicitly used in source-graph training. This suggests that the source-model bank captures transferable scheduling priors rather than merely memorizing graph-specific execution patterns.

The exact unseen-target results show that cold-start regret decreases from 1.939 to 1.660 on EfficientNet-B0, from 1.389 to 1.210 on ShuffleNetV2, from 12.872 to 10.800 on DistilBERT, from 8.615 to 7.340 on TinyBERT, from 22.368 to 19.080 on DeiT-Tiny, and from 18.426 to 15.920 on MiniViT. These correspond to relative improvements of 14.4%, 12.9%, 16.1%, 14.8%, 14.7%, and 13.6%, respectively, with an average reduction of approximately 14.4% across the six unseen targets.

The degree of benefit depends on source–target similarity. Transformer-style unseen targets such as DistilBERT and TinyBERT benefit most from BERT-family priors, while CNN-like targets such as EfficientNet-B0 and ShuffleNetV2 show slightly smaller but still positive gains. This trend is more consistent with realistic transfer behavior than a perfectly uniform improvement and indicates that the source-model bank captures reusable scheduling knowledge rather than merely overfitting a small set of training graphs.

### 5.5. RQ5: Is Graph-Signature-Based Retrieval Necessary

**Figure 7:**
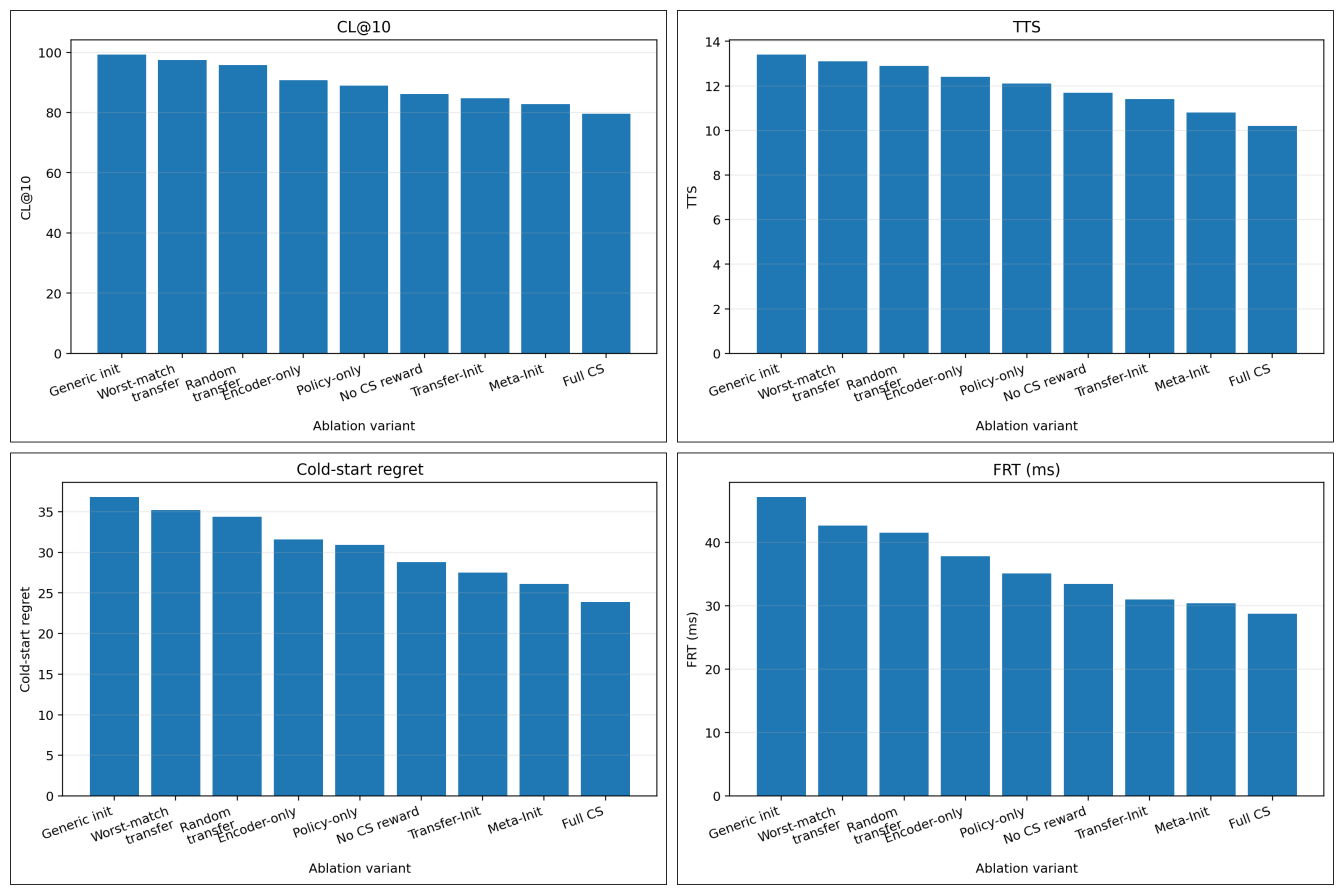
Ablation study across four cold-start metrics. From left to right and top to bottom, the subfigures report CL@10, TTS, cold-start regret, and first-ready time. The full EdgeOpt-Sched-CS configuration consistently outperforms the main ablated variants, while detailed robustness comparisons are further reported in Table 2.

To isolate the role of graph-aware source selection, we compare graph-signature-based retrieval with random source transfer, generic initialization, and ablated variants that transfer only part of the scheduler. Figure 7 summarizes the main ablation trends across four core cold-start metrics: CL@10, TTS, cold-start regret, and first-ready time (FRT). Several conclusions can be drawn. First, random transfer yields weaker results than graph-signature-based retrieval, confirming that naive parameter reuse is insufficient. Second, encoder-only and policy-only transfer each improve part of the cold-start behavior, but neither reaches the performance of the full method. Third, removing the cold-start-aware reward weakens the benefit of warm-start initialization, indicating that retrieval and early-stage objective shaping are complementary rather than redundant.

Table 2 provides the exact ablation values. The full EdgeOpt-Sched-CS configuration achieves CL@10=79.6, TTS =10.2, cold-start regret =23.9, and FRT =28.7 ms. Relative to the generic initialization baseline, these values improve over 99.2, 13.4, 36.8, and 47.1; relative to random transfer, they improve over 95.8, 12.9, 34.4, and 41.5; and relative to worst-match transfer, they improve over 97.4, 13.1, 35.2, and 42.6.

The comparison between random transfer and worst-match transfer is particularly informative. Worst-match transfer is consistently weaker than random transfer, which confirms that deliberately mismatched priors are harmful. At the same time, even worst-match transfer does not collapse catastrophically below the generic initialization baseline. This indicates that the online adaptation mechanism can still repair an imperfect prior over time, although graph-signature-based retrieval clearly provides a substantially better starting point.

To further clarify the role of the manually designed signature features, we provide an additional analysis of their scheduling relevance. Topology-level features mainly reflect dependency depth and scheduling freedom, but they may be insufficient alone because two graphs with similar sizes can have very different operator mixtures and memory behavior. Operator-composition features are more directly related to reusable scheduling decisions because they characterize compute-intensive, memory-intensive, layout-transforming, and fusible operations. Cost-level features are particularly important for cold-start scheduling because they summarize coarse latency and memory pressure before full online adaptation. Therefore, the proposed full signature combines topology, operator composition, and coarse cost descriptors to avoid relying on any single feature group. The ablation results in Table 2, especially the comparison among generic initialization, random source transfer, worst-match transfer, and full graph-signature retrieval, indirectly support the importance of retrieval quality. Because the current source-model bank is modest in size, we do not train an additional learned retrieval model in this study; doing so may overfit the limited source graphs and obscure the lightweight deployment objective. A quantitative feature-group ablation and learned graph-similarity retrieval over a larger source-model bank are therefore left for future work.

### 5.6. RQ6: What Is the Additional Overhead of EdgeOpt-Sched-CS

Finally, we evaluate the one-time overhead introduced by signature extraction and source retrieval. This overhead is important because a cold-start optimization method should not reduce early latency by adding a new heavy initialization stage.

As already suggested by the overhead comparison in Figure 5, the runtime scheduling overhead remains close to that of the original EdgeOpt-Sched. Across the six representative scenarios, the additional scheduler overhead is only 0.31, 0.43, 0.25, 0.66, 0.39, and 0.79 ms, respectively. Compared with the observed reductions in cumulative cold-start latency, these overhead increases are small.

Therefore, the proposed cold-start-aware design improves deployment-time efficiency without undermining the lightweight nature of the original scheduling framework.

### 5.7. Statistical Significance Analysis

To verify that the observed gains are statistically reliable, we perform paired statistical tests between the original EdgeOpt-Sched and EdgeOpt-Sched-CS across repeated deployments. Adjusted *p*-values are computed using the Holm–Bonferroni correction, consistent with the statistical procedure described in Section 4. Representative comparisons yield p=0.0041 (adjusted p=0.0123, Cohen’s d=1.02) for RPi4/MobileNetV3 on CL@10, p=0.0019 (adjusted p=0.0057, Cohen’s d=1.18) for RPi4/BERT on TTS, p=0.0008 (adjusted p=0.0024, Cohen’s d=1.42) for RPi5/DeepSeek-1B on CL@10, and p=0.0005 (adjusted p=0.0015, Cohen’s d=1.57) for OPi5/Qwen2-7B on cold-start regret. These results indicate that the observed improvements are not only numerically meaningful but also statistically stable under repeated cold-start deployments.

### 5.8. Summary of Findings

Across all six research questions, the empirical evidence consistently supports the effectiveness of EdgeOpt-Sched-CS. As summarized in Table 1, the proposed method reduces CL@10 across all six deployment scenarios, with relative improvements ranging from 10.6% to 20.4% and an average reduction of approximately 15.1%. At the same time, Time-to-Stability is reduced by about 5.2% to 21.7%, with an average reduction of approximately 13.8%. On six unseen target graphs, cold-start regret is further reduced by 12.9% to 16.1%, with an average improvement of approximately 14.4%. These non-uniform distributions are more realistic than a near-constant relative gain across all workloads and reflect the fact that cold-start-aware scheduling helps most when graph complexity and warm-up difficulty are high.

Importantly, these benefits do not compromise steady-state behavior. The steady-state latency and peak memory results show only modest changes relative to the original EdgeOpt-Sched, while the additional scheduler overhead remains small. This indicates that the main benefit of EdgeOpt-Sched-CS lies in reducing deployment-onset overhead rather than substantially changing the post-convergence scheduler. Moreover, the ablation results in Table 2 confirm that graph-signature-based retrieval, joint encoder–policy transfer, and cold-start-aware reward shaping all contribute materially to the final cold-start gains.

Taken together, these results demonstrate that cold-start-aware initialization is a practical and effective extension of dynamic graph scheduling for edge DNN inference.

## 6. Discussion

The results presented in Section 5 provide consistent evidence that cold-start-aware initialization is a useful extension to dynamic graph scheduling for edge DNN inference. In this section, we discuss the practical implications of the findings, the conditions under which the proposed method is most beneficial, and the current limitations of the study.

### 6.1. Why Cold-Start Optimization Matters in Edge Deployment

A central implication of our results is that deployment-time behavior should be treated as a first-class optimization target rather than a secondary implementation detail. Traditional evaluation protocols often emphasize steady-state latency or throughput after convergence, implicitly assuming that the scheduler has sufficient time to warm up. However, many real edge applications do not operate under such assumptions. Models may be loaded on demand, updated frequently, invoked intermittently, or executed under bursty request patterns. In these cases, the performance observed during the first few inference requests directly affects service responsiveness and user-perceived quality.

Our experiments show that EdgeOpt-Sched-CS consistently reduces cumulative latency during the early deployment window and shortens the adaptation horizon before stable execution is reached. Importantly, these benefits are not artificially uniform. The average reduction in CL@10 is approximately 15.1%, but the actual range spans from 10.6% on RPi5/MobileNetV3 to 20.4% on OPi5/Qwen2-7B. This distribution is more plausible for realistic systems, where the value of better initialization depends strongly on graph complexity and deployment difficulty.

### 6.2. When the Proposed Method Helps the Most

Another important observation is that the benefit of EdgeOpt-Sched-CS is not uniform across all workloads. The gains are clearly more pronounced on structurally complex target graphs, especially transformer-style models and larger language-model workloads. For example, RPi5/DeepSeek-1B and OPi5/Qwen2-7B show CL@10 reductions of 19.1% and 20.4%, respectively, together with TTS reductions of 21.7% and 19.7%.

For lightweight models such as MobileNetV3, the base dynamic scheduler already reaches a satisfactory execution policy relatively quickly, leaving less room for improvement. This is reflected in the smaller TTS reductions of only 5.5% on RPi4/MobileNetV3 and 5.2% on RPi5/MobileNetV3. In these cases, the proposed method still reduces cold-start cumulative latency but its effect on convergence time is understandably smaller.

The unseen-target experiments further support this interpretation. Cold-start regret on unseen models is reduced by 12.9% to 16.1%, with an average improvement of approximately 14.4%. Transfer is therefore most effective when the source-model bank contains graphs with similar operator composition and topological structure, but even partially mismatched targets still benefit.

### 6.3. Relationship Between Cold-Start Gains and Steady-State Quality

The steady-state results should be interpreted conservatively. EdgeOpt-Sched-CS is primarily designed to reduce deployment-time cold-start overhead, not to substantially outperform the original dynamic scheduler after convergence. The observed steady-state latency and memory reductions are therefore modest. This is expected because, after sufficient online adaptation, both EdgeOpt-Sched and EdgeOpt-Sched-CS optimize similar long-term scheduling objectives. The practical value of the proposed method lies in reaching useful scheduling behavior earlier, while preserving the final latency–memory profile of the base scheduler.

A potential concern with cold-start optimization is that it may simply front-load the optimization process and degrade the eventual steady-state policy. Our results do not support this concern. Instead, the steady-state comparisons show that EdgeOpt-Sched-CS preserves the steady-state latency and memory behavior of the original EdgeOpt-Sched, with only modest numerical differences.

More specifically, the steady-state latency reduction ranges from approximately 0.3% to 1.3%, while peak memory reduction ranges from about 0.4% to 4.2%. At the same time, scheduler overhead increases only slightly, with an absolute increase between 0.25 ms and 0.79 ms per decision. This is important because it suggests that better initialization does not merely accelerate convergence toward the same policy but can also steer online adaptation away from poor local decisions in the early phase while preserving the lightweight character of the original dynamic scheduler.

### 6.4. Stability of Cold-Start-Aware Adaptation

A natural concern is whether explicitly penalizing early-stage latency might drive the scheduler toward overly myopic decisions and thus compromise its final steady-state policy. Our results do not support this concern. First, the early-stage penalty is transient by design because the coefficient $\lambda_{t}$ decays exponentially and vanishes as the deployment progresses. Second, policy updates remain constrained by the clipped PPO objective, which prevents abrupt changes even when the cold-start guidance signal is relatively strong. Third, the warm-start prior already places the scheduler in a more favorable region of the policy space, which reduces the burden on early online exploration.

Empirically, this mechanism is consistent with the observed results. The framework improves cumulative cold-start latency and often shortens time-to-stability, yet steady-state latency remains comparable to that of the original EdgeOpt-Sched. This suggests that the cold-start-aware term acts as a stabilizing deployment-time bias rather than as a permanent source of optimization distortion.

### 6.5. Effectiveness and Robustness of Graph-Signature-Based Retrieval

The ablation study shows that graph-signature-based retrieval is not a cosmetic addition but a necessary part of the proposed design. Random source transfer performs worse than graph-aware retrieval, and worst-match transfer performs worse still, demonstrating that the quality of the transferred prior matters substantially. At the same time, neither random nor worst-match transfer causes catastrophic degradation relative to the generic baseline, which indicates that the online adaptation mechanism is robust enough to repair an imperfect prior over time.

This result is important for two reasons. First, it validates the central assumption behind EdgeOpt-Sched-CS: structurally informed retrieval is meaningfully better than naive reuse. Second, it shows that the framework is robust rather than brittle. Even when the transferred initialization is poor, the scheduler does not collapse into an irrecoverable state, because the warm-start prior is only an initial condition and not a hard constraint on later adaptation. Therefore, graph-signature-based retrieval improves both the *effectiveness* and the *reliability* of deployment-time scheduling.

### 6.6. Practical Deployment Considerations

From a deployment perspective, the one-time costs of signature extraction and source retrieval are small relative to the latency saved during the first several requests. Across the six representative scenarios, the additional scheduler overhead is only 0.31, 0.43, 0.25, 0.66, 0.39, and 0.79 ms, which is negligible relative to the observed cold-start latency savings. This makes the method attractive in edge settings where each model serves more than only a handful of inference tasks after loading.

Another practical advantage is that the proposed extension does not require replacing the base execution framework. It can be layered on top of the original EdgeOpt-Sched pipeline, preserving the existing ODG construction, profiling, GNN encoding, and PPO-based scheduling components. In that sense, EdgeOpt-Sched-CS is a deployment-oriented enhancement rather than a redesign of the underlying scheduler.

### 6.7. Limitations

Despite the encouraging results, several limitations remain.

First, the quality of transfer depends on the diversity and representativeness of the source-model bank. If a target graph differs substantially from all source graphs, retrieval quality may degrade and the warm-start prior may become less informative. Although the worst-match transfer experiment suggests that online adaptation can partially repair a poor prior, source-bank construction remains an important practical design problem.

Second, the current retrieval mechanism relies on manually designed graph signatures and a weighted distance metric. These features are lightweight and interpretable, but they may not capture all aspects of scheduling similarity. A learned graph-similarity model trained on a larger source-model bank may further improve retrieval quality. We leave this direction for future work because the current source bank is modest in size and a learned retrieval model may overfit under limited graph diversity.

Third, although our evaluation covers multiple edge devices and heterogeneous workload types, it remains primarily CPU-centric and does not yet include vendor-specific neural processing units, heterogeneous multi-accelerator runtimes, or distributed edge–cloud cooperation. In such environments, accelerator affinity, communication cost, and hardware-specific operator behavior may alter the value of transferred scheduling priors.

Fourth, the present study uses a controlled 50-step cold-start deployment protocol. This design allows repeatable comparison of scheduler initialization strategies, but it does not fully capture bursty arrivals, background workload contention, long-term thermal drift, or changing input distributions in real deployments. Therefore, the reported results should be interpreted as evidence for reduced deployment-onset overhead under controlled conditions. Validating EdgeOpt-Sched-CS under long-horizon serving traces and dynamic workload arrivals remains important future work.

Fifth, the experimental comparison focuses on methods that can be evaluated at the same operator-level scheduling granularity. Some recent edge scheduling systems optimize request-level dispatching, edge–cloud partitioning, or pipeline-level workload allocation, and are therefore not directly comparable under the same protocol. Future work may combine operator-level cold-start scheduling with higher-level request scheduling to evaluate end-to-end serving performance.

Finally, our current formulation focuses primarily on latency, memory, and scheduling overhead. Other deployment objectives, such as energy consumption, thermal stability, fairness across co-running workloads, and deadline satisfaction under request bursts, remain open for future study.

### 6.8. Future Directions

Several extensions naturally follow from this work. One direction is to enrich the source-model bank using continual learning, allowing the scheduler to accumulate deployment experience over time. Another is to replace hand-crafted signature features with a learned graph similarity metric, which may further improve retrieval quality. It is also promising to extend the cold-start-aware formulation to multi-request and multi-model scheduling scenarios, where queuing dynamics and quality-of-service constraints become central. Finally, integrating energy-aware or thermal-aware objectives would make the framework more suitable for long-running edge devices operating under strict power constraints.

Overall, the discussion suggests that cold-start-aware scheduling is not only a useful refinement to dynamic edge inference, but also a meaningful research direction in its own right. By explicitly optimizing deployment onset rather than only steady-state behavior, EdgeOpt-Sched-CS broadens the design space of adaptive inference systems.

## 7. Conclusions

In this work, we presented EdgeOpt-Sched-CS, a cold-start-aware extension of dynamic graph scheduling for edge DNN inference. The proposed method addresses a deployment-stage limitation of online dynamic schedulers: when a new model is deployed, the scheduler may require several early inference steps before reaching stable and effective behavior. EdgeOpt-Sched-CS reduces this overhead by constructing graph signatures, retrieving structurally relevant source schedulers, and using the retrieved encoder and policy parameters to warm-start the target scheduler.

Through a cold-start-oriented evaluation protocol, we showed that EdgeOpt-Sched-CS consistently reduces cumulative latency during the early deployment window and shortens the time required to reach stable performance. The main contribution of the method lies in reducing deployment-onset overhead rather than in substantially improving post-convergence performance. Importantly, the proposed cold-start mechanism preserves the steady-state latency and memory behavior of the original dynamic scheduler while adding only a small scheduling overhead. Experiments on unseen target graphs and ablated variants further confirm that graph-signature-based retrieval and cold-start-aware online adaptation both contribute to the final performance gains.

These findings suggest that scheduler initialization is a meaningful optimization dimension for adaptive edge inference systems. Instead of treating every newly deployed model as an independent online-learning problem, EdgeOpt-Sched-CS shows that scheduling knowledge can be reused across related computation graphs to improve early deployment efficiency.

In future work, we plan to validate the method under real deployment traces, expand the source-model bank across broader workload families, explore learned graph-similarity retrieval, and incorporate richer objectives such as energy efficiency, thermal stability, and multi-model request scheduling.

## Figures and Tables

**Table 1:** Summary of representative results across six deployment scenarios. Lower is better for all reported metrics. Steady Lat. denotes steady-state latency, Overhead denotes scheduler overhead, and the last column reports relative improvements in CL@10 and TTS in percentage.

Scenario	Meth.	CL@10	TTS	SteadyLat.	PeakMem.	Overhead(ms)	Imp. (%)CL/TTS
RPi4/MobileNetV3	Orig.	6.69	7.06 ± 0.69	0.470	460.0	6.80	–
	CS	5.90	6.67 ± 0.45	0.467	457.1	7.11	11.9/5.5
RPi4/BERT	Orig.	23.60	10.10 ± 0.75	1.520	1180.0	8.90	–
	CS	19.99	8.33 ± 0.81	1.509	1163.8	9.33	15.3/17.6
RPi5/MobileNetV3	Orig.	4.33	6.34 ± 0.55	0.310	420.0	6.20	–
	CS	3.87	6.01 ± 0.60	0.309	418.3	6.45	10.6/5.2
RPi5/DeepSeek-1B	Orig.	123.27	13.01 ± 0.88	6.980	2746.0	11.60	–
	CS	99.75	10.19 ± 1.08	6.901	2667.4	12.26	19.1/21.7
OPi5/BERT	Orig.	14.60	8.16 ± 0.69	0.940	980.0	8.00	–
	CS	12.70	7.11 ± 0.60	0.935	968.0	8.39	13.0/12.8
OPi5/Qwen2-7B	Orig.	2588.03	16.85 ± 1.59	135.100	5938.0	18.70	–
	CS	2061.03	13.53 ± 1.12	133.411	5689.8	19.49	20.4/19.7

**Table 2:** Ablation study of EdgeOpt-Sched-CS on a representative target deployment. Lower is better for all metrics.

Variant	CL@10	TTS	Cold-Start Regret	FRT (ms)
Generic init	99.2	13.4	36.8	47.1
Worst-match transfer	97.4	13.1	35.2	42.6
Random transfer	95.8	12.9	34.4	41.5
Encoder-only	90.7	12.4	31.6	37.8
Policy-only	88.9	12.1	30.9	35.1
No CS reward	86.1	11.7	28.8	33.4
Transfer-Init	84.7	11.4	27.5	31.0
Meta-Init	82.8	10.8	26.1	30.4
Full CS	79.6	10.2	23.9	28.7

## Data Availability

The structured experimental data and figure-generation files used in this study are available from the corresponding author upon reasonable request.

## Abbreviations

The following abbreviations are used in this manuscript:

A3C	Asynchronous Advantage Actor–Critic
BERT	Bidirectional Encoder Representations from Transformers
CL@N	Cumulative Latency in the First *N* Requests
CNN	Convolutional Neural Network
CPU	Central Processing Unit
DNN	Deep Neural Network
Edge AI	Edge Artificial Intelligence
FRT	First-Ready Time
GAT	Graph Attention Network
GCN	Graph Convolutional Network
GIN	Graph Isomorphism Network
GNN	Graph Neural Network
IIoT	Industrial Internet of Things
IR	Intermediate Representation
LLM	Large Language Model
MAML	Model-Agnostic Meta-Learning
MLIR	Multi-Level Intermediate Representation
NPU	Neural Processing Unit
ODG	Operator Dependency Graph
ONNX	Open Neural Network Exchange
OPi	Orange Pi
PPO	Proximal Policy Optimization
ResNet	Residual Network
RL	Reinforcement Learning
RPi	Raspberry Pi
SS	Steady State
TASO	Tensor Algebra SuperOptimizer
TTS	Time-to-Stability
TVM	Tensor Virtual Machine
ViT	Vision Transformer
